# Activity of roniciclib in medullary thyroid cancer

**DOI:** 10.18632/oncotarget.25555

**Published:** 2018-06-15

**Authors:** Shu-Fu Lin, Jen-Der Lin, Chuen Hsueh, Ting-Chao Chou, Richard J. Wong

**Affiliations:** ^1^ Department of Internal Medicine, Chang Gung Memorial Hospital, Chang Gung University, Taoyuan, Taiwan; ^2^ Department of Pathology, Chang Gung Memorial Hospital, Taoyuan, Taiwan; ^3^ Laboratory of Preclinical Pharmacology Core, Memorial Sloan-Kettering Cancer Center, New York, NY, USA; ^4^ Current address: PD Science, Inc., Paramus, NJ, USA; ^5^ Department of Surgery, Memorial Sloan-Kettering Cancer Center, New York, NY, USA

**Keywords:** roniciclib, cyclin-dependent kinase, medullary thyroid cancer

## Abstract

Altered cyclin-dependent kinase activity is observed in many human malignancies. Cyclin-dependent kinases that promote cell cycle progression may be promising targets in the treatment of cancer. The therapeutic effects of roniciclib, a cyclin-dependent kinase inhibitor for medullary thyroid cancer were investigated in the present study. Roniciclib inhibited medullary thyroid cancer cell proliferation in a dose-dependent manner. Roniciclib induced caspase-3 activity and contributed to apoptosis. Cell cycle progression was arrested in the G2 phase. *In vivo*, roniciclib treatment retarded the growth of tumors of medullary thyroid cancer xenografts. In addition, roniciclib in combination with sorafenib was more effective than either single treatment in a xenograft model. No morbidity was observed in animals treated with single roniciclib therapy and combination treatment of roniciclib and sorafenib. These data provide a rationale for clinical assessment of using roniciclib in the treatment of patients with medullary thyroid cancer.

## INTRODUCTION

Thyroid cancer is the most common malignancy of the endocrine system, with an increasing incidence worldwide in the past few decades [1, 2]. Medullary thyroid cancer (MTC) originates from parafollicular C cells and accounts for 3-5% of all thyroid cancers [3]. Some MTC is sporadic (40-45%) and the remaining is hereditary MTC. *RET* proto-oncogene mutations are the dominant pathogenesis of MTC [4–6]. The clinical course of MTC can be indolent for years. However, aggressive MTC is associated with a high mortality rate [7]. Two multi-kinase inhibitors, cabozantinib and vandetanib, improve progression-free survival and have recently been approved by the U.S. Food and Drug Administration for the treatment of progressive MTC [8, 9]. Nevertheless, both drugs have limited therapeutic efficacy in many patients and are associated with toxic effects that usually lead to interruption of treatment. Novel treatments with distinct therapeutic mechanisms are pivotal for improving the outcomes of patients with progressive MTC.

Malignancy is featured by uncontrolled cell proliferation resulting from genetic mutations in genes encoding cell cycle protein or upstream signaling pathways of cell cycle [10]. There are four distinct cell cycle phases (G0/G1, S, G2 and M). Cell cycle progression is regulated by multiple cell cycle-associated proteins, including cyclin-dependent kinases (CDKs). Specific CDKs are activated in complexes with their cyclin partners [10–12]. The formation of CDK4/6-cyclin D complexes contributes to cell cycle entry (from the quiescence G0 phase to G1 phase). These complexes also sequester p21 and p27, two CDK inhibitors, activate CDK2-cyclin E complex, and propel G1 to S phase transition. The CDK1/2-cyclin A complexes further ensure cell cycle progression through S to G2 phase. Finally, the activation of CDK1-cyclin B1 complex is required for mitotic entry and mitotic progression. Aberrant CDK activity is characterized in many human cancer types [13]. In addition to these canonical roles of CDKs, some data suggest that mammalian CDKs have other important cellular functions, including transcription and control of cell death [12]. For instance, CDK5 is pivotal for brain development, neuronal survival, and regulates cell proliferation of MTC [14, 15]. CDK7 is involved in phosphorylation of C-terminal repeat domain, an extension appended to the C terminus of a subunit of RNA polymerase II [16]. Inhibition of CDK7 suppresses oncogenic transcription and leads to tumor regression in a *MYCN*-driven neuroblastoma mouse model [17]. CDK9 is an important component of the positive transcription elongation factor b complex that is required for the proliferation of oncogenic *MYC*-driven hepatocellular carcinoma [18]. Given the pivotal biologic roles of CDKs in cell cycle progression and cell survival, targeting CDKs may be a promising therapeutic approach for cancer therapy.

Roniciclib is a potent inhibitor of cell-cycle CDKs (CDK1, CDK2, CDK3 and CDK4), transcriptional CDKs (CDK5, CDK7 and CDK9) and non-CDK kinases (including Aurora A) at IC_50_ values of ≤ 100 nmol/L [19]. Roniciclib arrests cell cycle progression, activates caspase-3 activity, and induces apoptosis *in vitro*. In mice, oral administration of roniciclib results in rapid absorption and exhibits potent efficacy in inhibiting tumor growth of cervical and lung tumor xenografts, with promising safety profiles [19]. This data suggests that roniciclib is a potential drug in the treatment of patients with malignancy.

In this study, we evaluated the therapeutic effects of roniciclib in MTC *in vitro* and *in vivo*. Roniciclib induces cytotoxicity in two MTC cell lines *in vitro*. *In vivo* study using a MTC xenograft model demonstrates significant therapeutic effect and promising safety.

## RESULTS

### Cytotoxicity of roniciclib in MTC cell lines

**Figure 1:**
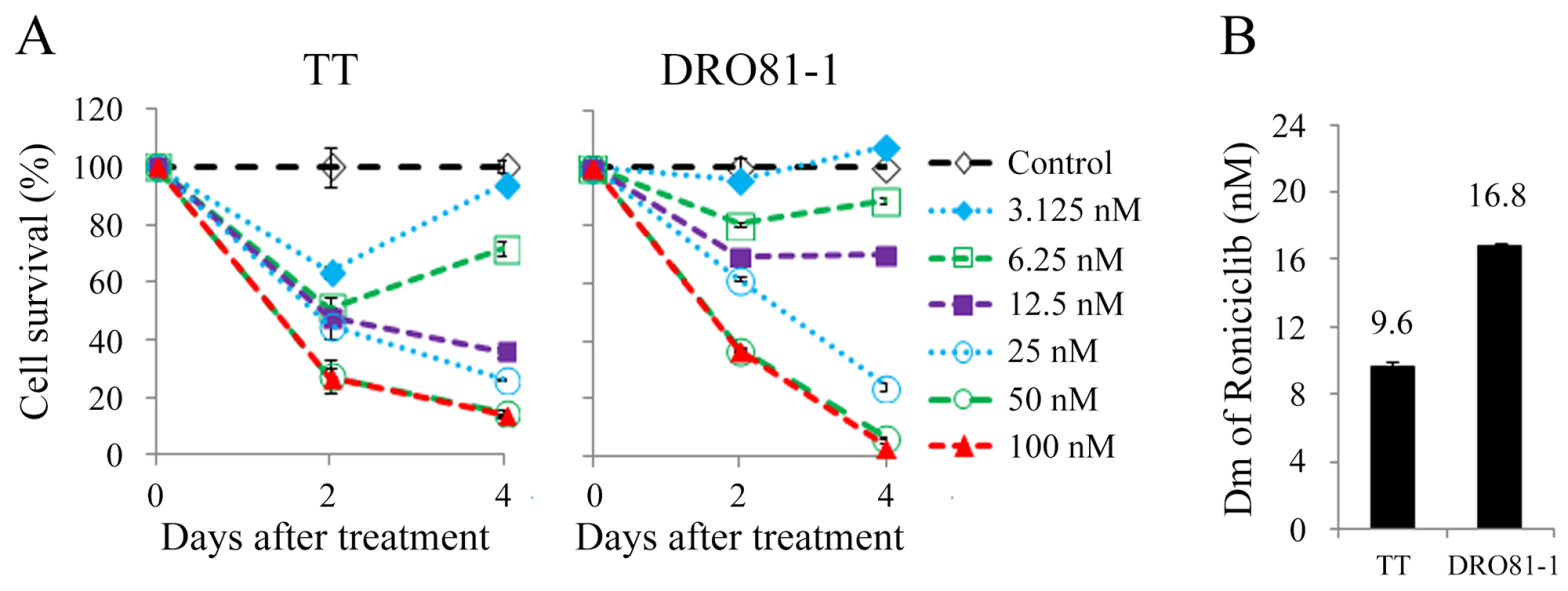
Roniciclib induces cytotoxicity in MTC cells **(A)** Cytotoxicity was evaluated in cells treated with a series of six two-fold dilutions of roniciclib starting from 100 nmol/L. Dose-response curves were obtained on days 2 and 4 using LDH assays. **(B)** Median-effect dose (Dm) of roniciclib on day 4 was calculated for each cell line using CompuSyn software.

Roniciclib inhibited cell survival in two MTC cell lines in a dose-dependent manner (Figure 1A). Most doses studied inhibited cell survival, and higher doses of roniciclib (≥ 25 nmol/L) revealed more durable effects and induced cytotoxicity over a 4-day treatment course in these two cell lines. Roniciclib at 25 nmol/L inhibited 73.3% (TT) and 75.7% (DRO81-1) of cell growth by day 4. At 100 nmol/L, roniciclib arrested > 86% of cell growth in TT and DRO81-1 cells. The potency of cytotoxicity of roniciclib in MTC cell lines was determined using CompuSyn software [20, 21]. The median-effect dose (Dm) was determined on day 4 (Figure 1B). TT cells had lower Dm (9.6 ± 0.3 nmol/L) than that of DRO81-1 cells (16.8 ± 0.2 nmol/L). These cytotoxic effects of roniciclib in two MTC cell lines were validated by counting viable cells using a microscope after a 4-day therapy (Supplementary Figure 1).

### Roniciclib induced apoptosis in MTC cell lines

**Figure 2:**
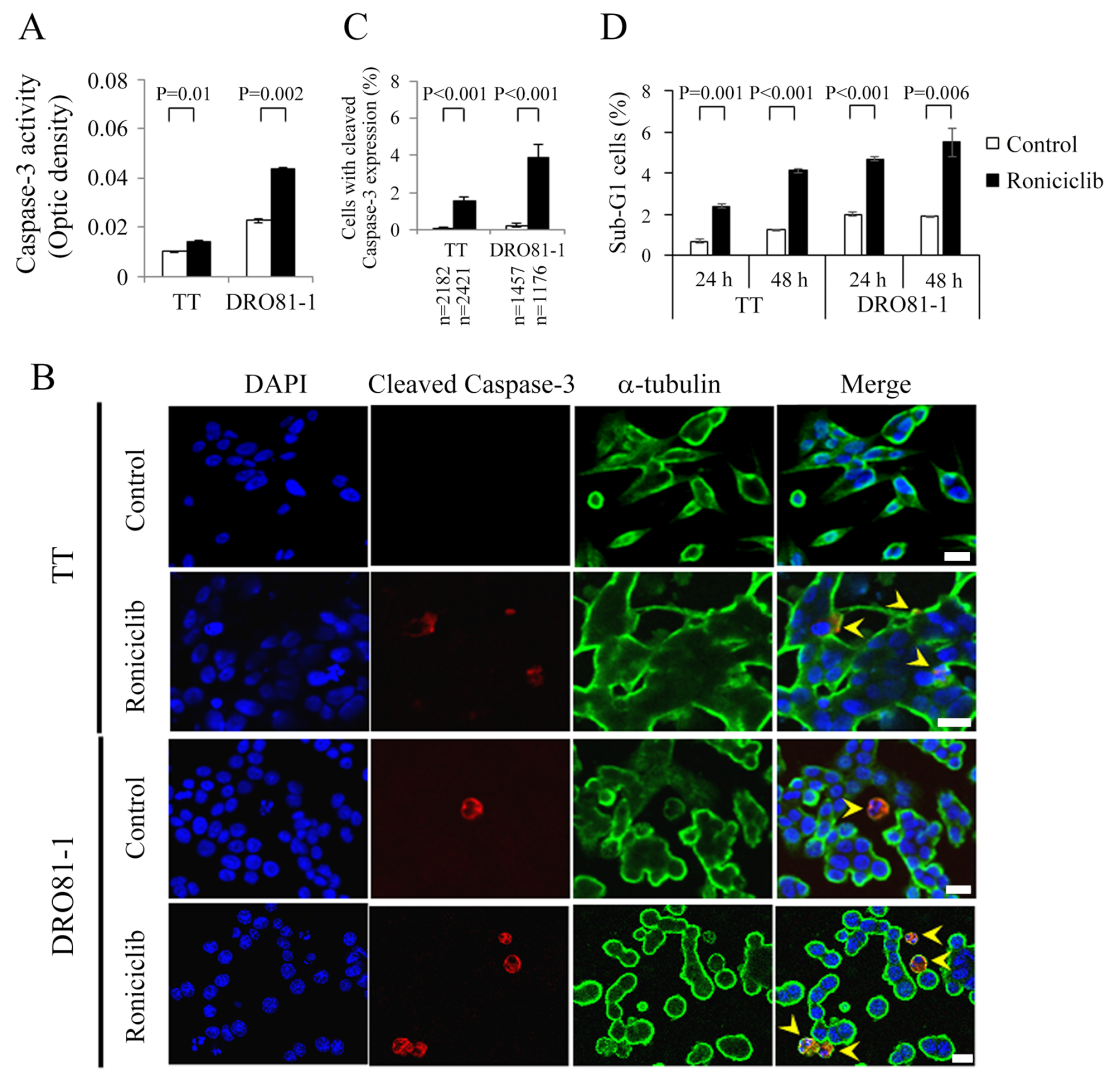
Roniciclib stimulates caspase-3 activity and causes apoptosis in MTC cells **(A)** Caspase-3 activity was evaluated using a fluorometric assay kit in cells treated with roniciclib (100 nmol/L) or vehicle for 24 h in TT and DRO81-1 cells. **(B)** Cells were treated with roniciclib (100 nmol/L) or placebo for 24 h and stained with fluorescent antibodies against DAPI (blue), cleaved caspase-3 (red) and α-tubulin (green). Cells with cleaved caspase-3 expression are identified (arrowhead). **(C)** The percentages of cells with cleaved caspase-3 expression were assessed after treatment with placebo or roniciclib (100 nmol/L) for 24 h. Cells were stained with cleaved caspase-3 and its expression was evaluated using immunofluorescence confocal microscopy. A minimum of 1176 cells was counted for each condition. Roniciclib significantly increased the proportion of cells with cleaved caspase-3 expression in TT and DRO81-1 cell lines. **(D)** Sub-G1 apoptotic cells were detected by measuring the DNA content using flow cytometry in cells treated with roniciclib (100 nmol/L) or vehicle for 24 h and 48 h. Roniciclib increased the proportion of sub-G1 cells in the two MTC cell lines. Scale bar, 20 μm.

Apoptosis, a process of programmed cell death, has a potential role in the treatment of cancer [22]. Roniciclib activates caspase-3 activity and induces apoptosis in HeLa cell line [19]. We evaluated the effects of roniciclib on apoptosis in two MTC cell lines. The effects of roniciclib (100 nmol/L) on caspase-3 activity were determined using a fluorometric assay at 24 h in TT and DRO81-1 cell lines (Figure 2A). Roniciclib significantly increased caspase-3 activity compared to control treatment in TT (0.014 ± 0.000-optical density [OD] and 0.01 ± 0.000-OD, P = 0.01) and DRO81-1 (0.044 ± 0.001-OD and 0.023 ± 0.001-OD, P = 0.002), demonstrating activation of caspase-3. Caspase-3 activation was also assessed by detection of cleaved caspase-3 (active form of caspase-3) using immunofluorescent analysis in two MTC cell lines (Figure 2B). The percentages of cells with cleaved caspase-3 expression were analyzed (Figure 2C). Roniciclib (100 nM for 24 h) significantly increased the proportions of cells with cleaved caspase-3 expression in TT (1.5 ± 0.2% and 0.1 ± 0.1%, P < 0.001) and DRO81-1 (3.9 ± 0.7% and 0.2 ± 0.1%, P < 0.001) when compared with control. Activation of caspase-3 may contribute to apoptotic cell death.

The ability of roniciclib to induce sub-G1 apoptosis in MTC cell lines was evaluated (Supplementary Figure 2). TT and DOR81-1 cell lines were exposed to roniciclib (100 nmol/L) and the proportion of sub-G1 apoptotic cells was calculated (Figure 2D). Roniciclib significantly induced higher proportions of sub-G1 cells than did control treatment in TT at 24 h (2.4 ± 0.1% and 0.7 ± 0.1%, P = 0.001) and 48 h (4.1 ± 0.1% and 1.2 ± 0.0%, P < 0.001). Similar effects appeared in DRO81-1 at 24 h (4.7 ± 0.1% and 2.0 ± 0.1%, P < 0.001) and 48 h (5.5 ± 0.7% and 1.9 ± 0.1%, P = 0.006). These data reveal that roniciclib induces apoptosis in MTC cells. The ability of roniciclib to induce apoptosis was confirmed by assessing the expression of cleaved poly (ADP-ribose) polymerase (PARP), a marker of apoptosis, and analyzing the proportions of early apoptotic cells in TT and DRO81-1 cell lines treated with roniciclib (100 nmol/L) or placebo (Supplementary Figure 3).

### Effects of roniciclib on the cell cycle

**Figure 3:**
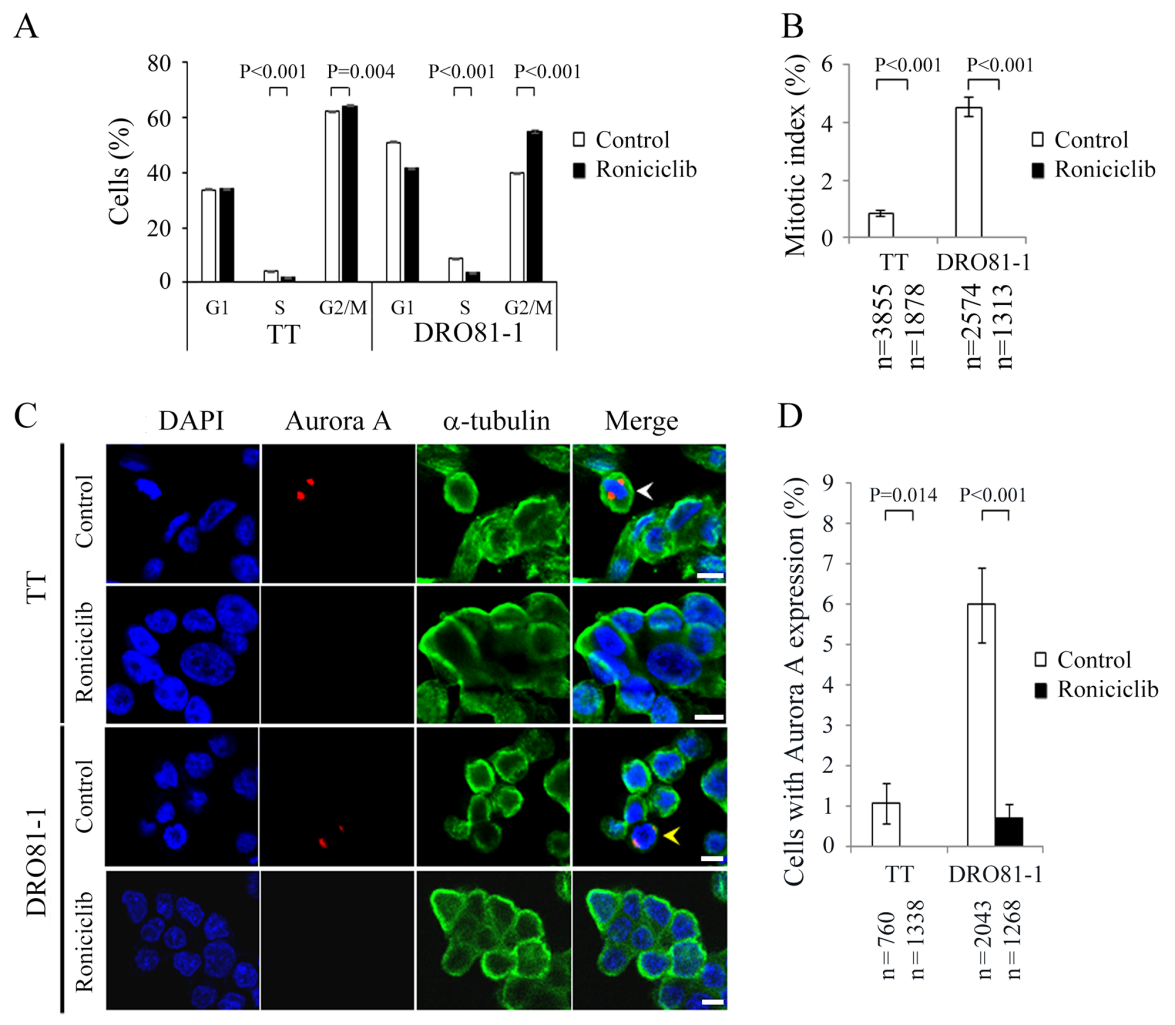
Roniciclib decreases the level of Aurora A and accumulates cells in G2 phase in MTC cells **(A)** Cell cycle distribution was analyzed by evaluating the DNA content using flow cytometry in TT and DRO81-1 cells treated with placebo or roniciclib (100 nmol/L) for 24 h. Statistical analyses revealed that roniciclib (100 nmol/L) decreased cell population in S phase and accumulated cells in G2/M phase at 24 h in TT and DRO81-1 cells. **(B)** The proportion of MTC cells in mitosis was assessed after treatment with roniciclib (100 nmol/L) or placebo for 24 h. Cells were stained with DAPI and chromosome characteristics were evaluated using immunofluorescence confocal microscopy. Mitotic index was assessed with a minimum of 1313 cells and counted from at least ten different fields for each condition. Roniciclib significantly decreased the proportion of cells in mitosis in TT and DRO81-1 cell lines. **(C)** Cells were treated with roniciclib (100 nmol/L) or placebo for 24 h and stained with fluorescent antibodies against DAPI (blue), Aurora A (red) and α-tubulin (green). Placebo-treated cells in metaphase (white arrowhead) and anaphase (yellow arrowhead) are indicated. **(D)** The percentages of cells with Aurora A expression were assessed after treatment with placebo or roniciclib (100 nmol/L) for 24 h using immunofluorescence confocal microscopy. A minimum of 760 cells was counted for each condition. Roniciclib significantly decreased the proportion of cells with Aurora A expression in two MTC cell lines. Scale bar, 10 μm.

The effect of roniciclib (100 nmol/L for 24 h) on cell cycle distribution in two MTC cell lines was evaluated (Supplementary Figure 4). The cell cycle data was analyzed (Figure 3A). Compared with placebo treatment, roniciclib significantly induced cell accumulation in G2/M phase in TT (64.2 ± 0.3% and 62.1 ± 0.2%, P = 0.004) and DRO81-1 (55.1 ± 0.6% and 40.0 ± 0.1%, P < 0.001), demonstrating induction of G2/M arrest. In addition to G2/M phase arrest, roniciclib decreased cell population in S phase in TT (1.9 ± 0.1% and 4.1 ± 0.1%, P < 0.001) and DRO81-1 (3.6 ± 0.1% and 8.9 ± 0.1%, P < 0.001).

The ability of roniciclib to arrest cells in mitotic phase was determined using a confocal fluorescence microscope (Supplementary Figure 5). Mitotic cells were identified and the mitotic index was calculated for two thyroid cancer cell lines (Figure 3B). Compared with control treatment, roniciclib (100 nmol/L) treatment for 24 h significantly decreased the percentage of mitotic cells in TT (0.0 ± 0.0% and 0.8 ± 0.1%, P < 0.001) and DRO81-1 (0.0 ± 0.0% and 4.5 ± 0.3%, P < 0.001), demonstrating that roniciclib prevented MTC cell progression into mitosis. Roniciclib treatment for 48 h consistently inhibited mitotic entry in TT cells (Supplementary Figure 6).

Aurora A is essential for mitotic entry and mitotic progression [23, 24]. We evaluated the expression of Aurora A in TT and DRO81-1 cells treated with roniciclib (100 nmol/L) or placebo for 24 h by using confocal fluorescence microscopy (Figure 3C). The percentages of cells with Aurora A expression were analyzed (Figure 3D). Compared with control treatment, roniciclib significantly decreased the proportion of cells with Aurora A expression in TT (0.0 ± 0.0% and 1.1 ± 0.5%, P = 0.014) and DRO81-1 (0.7 ± 0.4% and 6.0 ± 0.9%, P < 0.001). These data demonstrate that roniciclib decreases Aurora A expression in MTC cells.

5-bromo-2’-deoxyuridine (BrdU) can be incorporated into newly synthesized DNA of dividing cells and is frequently used in the evaluation of cell proliferation. The effects of roniciclib on BrdU uptake in two MTC cell lines were assessed (Supplementary Figure 7). Compared with control treatment, roniciclib (100 nmol/L) significantly decreased BrdU uptake in TT (0.003 ± 0.000-OD and 0.006 ± 0.001-OD, P = 0.002) and DRO81-1 cells (0.009 ± 0.001-OD and 0.012 ± 0.000-OD, P = 0.039), suggesting inhibition of cell proliferation.

### Monotherapy with roniciclib for murine flank MTC tumors

**Figure 4:**
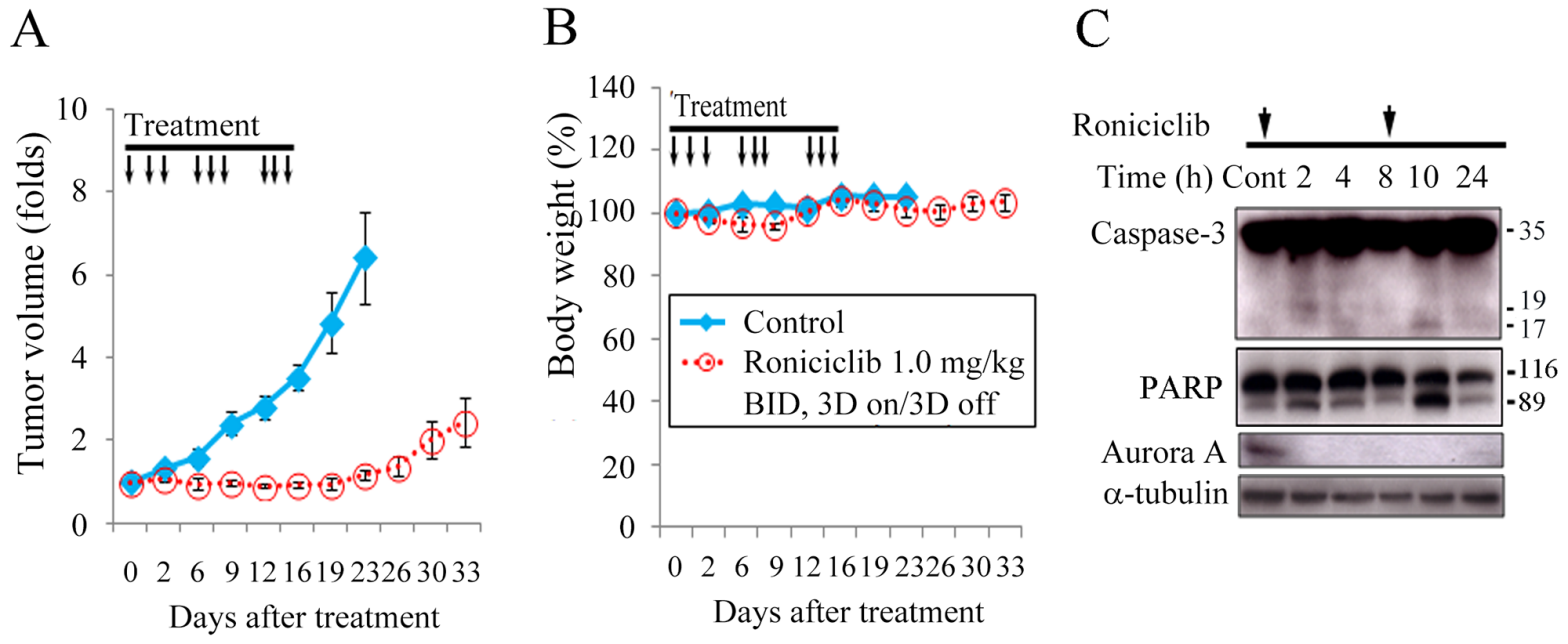
Roniciclib inhibits subcutaneous xenograft growth of a MTC model **(A)** The therapeutic efficacy of roniciclib was evaluated in mice bearing TT flank tumors. Serial oral gavage of roniciclib (1.0 mg/kg) significantly retarded TT tumor growth by day 6 compared with the control group (1.0-fold and 1.6-fold, P = 0.036), and the effect persisted through day 23. **(B)** Serial treatment of roniciclib slightly, but significantly induced body weight loss on day 9 (96.0% and 102.6%, P = 0.001) when compared with control mice. **(C)** The molecular effects of roniciclib (1.0 mg/kg) treatment were evaluated in TT tumors using western blot analysis. Arrow, roniciclib and placebo treatment.

Female nude mice bearing flank xenografts of TT were used to evaluate the therapeutic efficacy and safety of roniciclib *in vivo*. Animals with established TT flank tumors with a mean diameter of 4.5 mm were treated with oral gavage of placebo (*n* = 5) or roniciclib (1.0 mg/kg, *n* = 5) twice a day for three cycles of 3-day on and 3-day off therapy (Supplementary Figure 8). Relative tumor volume of each xenograft was calculated as *Vx*/*V1*, where *Vx* is the volume in mm^3^ at an indicated time and *V1* at the beginning of treatment (Figure 4A). Roniciclib significantly retarded TT tumor growth by day 6 compared with the control group (1.0-fold and 1.6-fold, P = 0.036), and the effect persisted through day 23 (1.2-fold and 6.4-fold, P = 0.002). Serial administration of roniciclib transiently and minimally resulted in significant body weight loss on day 9 (96.0% and 102.6%, P = 0.001) in mice bearing TT xenografts (Figure 4B).

The molecular effects of roniciclib treatment in TT xenografts (1.0 mg/kg) were evaluated (Figure 4C). The ratios of cleaved caspase-3 (active form of caspase-3), cleaved PARP and Aurora A to α-tubulin at each time point were calculated. Relative expression was analyzed using the control value as reference (Supplementary Figure 9). Roniciclib treatment rapidly (by 2 h) increased the levels of cleaved caspase-3 and cleaved PARP, and the effect was absent by 8 h. Aurora A level was decreased by 2 h, and the inhibitory effect persisted for 24 h.

### Interaction of roniciclib and sorafenib in MTC cells

Sorafenib has demonstrated efficacy in the treatment of patients with MTC and is suggested as a therapeutic option for vandetanib- and cabozantinib-refractory MTC [25, 26]. However, some patients are refractory to sorafenib treatment and strategies to improve sorafenib efficacy are mandatory. We evaluated the interactions between roniciclib and sorafenib in two MTC cell lines.

**Figure 5:**
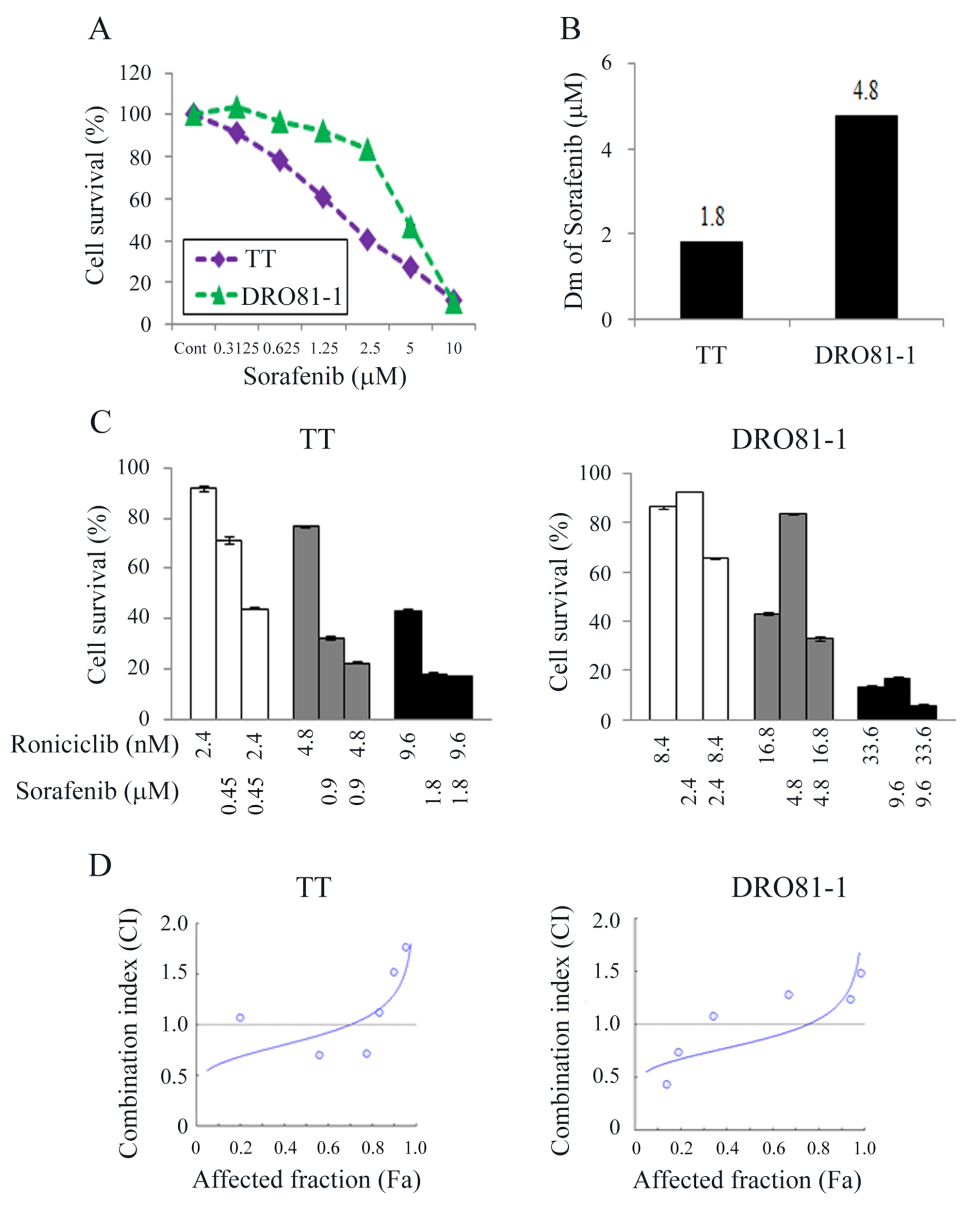
Combination therapy of roniciclib and sorafenib against MTC cells **(A)** Cytotoxicity was evaluated in cells treated with a series of six two-fold dilutions of sorafenib starting at 10 μmol/L in TT and DRO81-1 cells. Dose-response curves were obtained on day 4 using LDH assays. **(B)** Median-effect dose (Dm) of sorafenib on day 4 was calculated for each cell line using CompuSyn software. **(C)** The cytotoxic effects of roniciclib and sorafenib alone or in combination after a 4-day treatment in MTC cells were evaluated using LDH assays. **(D)** The combination index (CI) of roniciclib and sorafenib was calculated using CompuSyn software. Roniciclib and sorafenib had synergistic to antagonistic effects in TT and DRO81-1 cells. Synergistic effects were recognized when lower fractions of cells affected in TT (affected fraction ≤ 0.7) and DRO81-1 (affected fraction ≤ 0.75). Horizontal lines at CI = 1 were drawn for discrimination of synergism (CI < 1) and antagonism (CI > 1).

The cytotoxic effects of sorafenib in TT and DRO81-1 cells were studied (Figure 5A). Sorafenib inhibited cell proliferation in a dose-dependent manner in these cell lines. Sorafenib at 1.25 μmol/L inhibited 39.5% (TT) and 7.7% (DRO81-1) of cell growth by day 4. At 10 μmol/L, sorafenib arrested > 88% of cell growth in TT and DRO81-1 cells. The median effect dose (Dm) of sorafenib on day 4 was calculated for each cell line (Figure 5B). TT was more sensitive to sorafenib than DRO81-1 cell line (Dm; TT = 1.8 μmol/L, DRO81-1 = 4.8 μmol/L).

Interactions between roniciclib and sorafenib were evaluated in two MTC cell lines (Figure 5C). The combination of roniciclib and sorafenib improved cytotoxicity over single agent therapy in these cell lines, particularly at lower doses of treatment. The combination index (CI) of roniciclib and sorafenib was analyzed using the Chou-Talalay method, where CI < 1 indicates synergism, CI = 1 shows an additive effect, and CI > 1 indicates antagonism [20]. The combination of roniciclib and sorafenib ranged from synergistic to antagonist in TT and DRO81-1 (CI; 0.55-1.71 and 0.55-1.6, respectively) (Figure 5D). Synergistic effects appeared when lower proportions of cells were affected in TT (affected fraction ≤ 0.7) and DRO81-1 (affected fraction ≤ 0.75). Our data revealed that combination therapy of roniciclib and sorafenib were mostly synergistic in TT and DRO81-1 cell lines. Therefore, we evaluated the combination treatment effect of roniciclib and sorafenib in MTC *in vivo*.

### Combination therapy of roniciclib and sorafenib for murine flank MTC tumors

**Figure 6:**
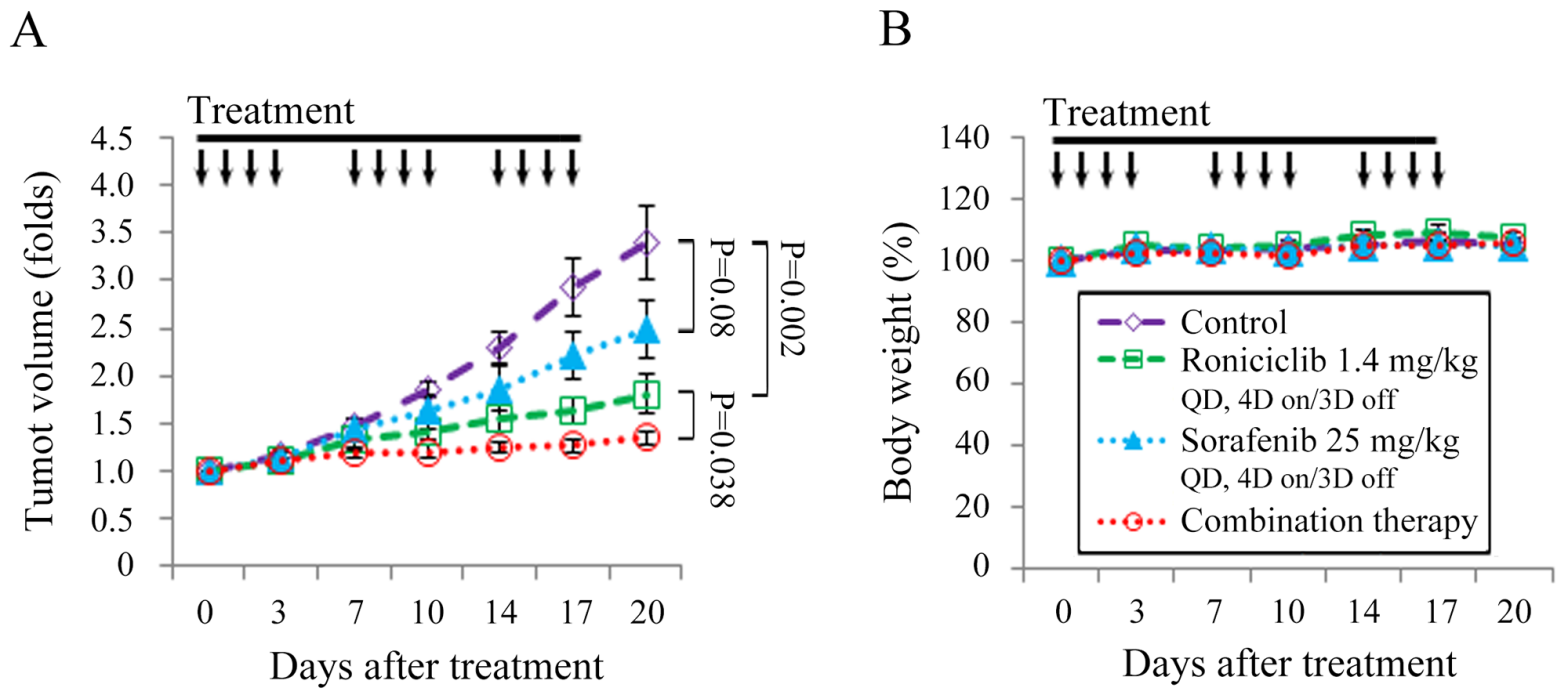
Combination of roniciclib and sorafenib therapy in a murine MTC xenograft tumor model **(A)** TT flank tumors were established in nude mice and treated with oral gavage of placebo, roniciclib (1.4 mg/kg), sorafenib (25 mg/kg) and combination therapy once a day for three cycles of 4-day on and 3-day off therapy. Tumor growth was significantly lower for mice treated with combination therapy compared with single modality treatment or control on day 17 and day 20. **(B)** No significant changes in body weight attributable to therapy with roniciclib, sorafenib, or combination therapy were observed compared with the placebo group. Arrow, placebo, roniciclib, sorafenib and combination treatment.

Animals with established TT flank tumors with a mean diameter of 4.4 mm were treated with oral gavage of placebo (*n* = 10), roniciclib (1.4 mg/kg, *n* = 9), sorafenib (25 mg/kg, *n* = 9) and combination therapy (*n* = 9) once a day for three cycles of 4-day on and 3-day off therapy (Supplementary Figure 10). Relative tumor volume of each xenograft was calculated (Figure 6A). The dose of sorafenib chosen was based on a previous report [27]. Roniciclib significantly retarded TT tumor growth by day 10 compared with the control group (1.4-fold and 1.9-fold, P = 0.002), and the effect persisted through day 20 (1.8-fold and 3.4-fold, P = 0.002). Sorafenib demonstrated a trend towards slower TT tumor growth after 20 days compared with the control mice, but this was not statistically significant (2.5-fold and 3.4-fold on day 20, P = 0.08). Combination therapy significantly retarded TT tumor growth on day 17 compared with the control group (1.3-fold and 2.9-fold, P < 0.001), roniciclib group (1.3-fold and 1.6-fold, P = 0.035), and the sorafenib group (1.3-fold and 2.2-fold, P = 0.002). The inhibitory effect of combination therapy persisted through day 20. Serial treatment of roniciclib, sorafenib, or combination therapy did not result in significant changes in body weight as compared with control treatment (Figure 6B).

## DISCUSSION

Roniciclib effectively inhibited cell proliferation with a relatively low median-effect dose (≤ 16.8 nmol/L) in two MTC cell lines. Roniciclib treatment effectively repressed tumor growth of a MTC xenograft model with promising safety profiles. We also evaluated the cytotoxicity of roniciclib in non-cancerous human umbilical vein endothelial cells (HUVEC; Supplementary Figure 11). The median-effect dose of roniciclib in HUVEC (24.5 nmol/L) was higher than that of MTC cell lines, suggesting the tumor selectivity of roniciclib.

Roniciclib treatment activated caspase-3 activity and caused apoptosis in MTC cell lines. Inhibition of caspase-3 activity decreased cytotoxicity of roniciclib treatment in MTC cells (Supplementary Figure 12). Though, this caspase-3 inhibition did not completely rescue cytotoxicity of roniciclib, indicating other mechanisms, such as proliferation arrest also contributed to the cytotoxicity. Apoptosis-mediated cell death is through two main pathways, extrinsic (death receptor) and intrinsic (mitochondrial) pathways. Multiple CDKs participate in the apoptosis signaling pathways [12]. Inhibition of CDK activity is, therefore, likely to contribute toward the apoptotic response. The underlying mechanisms of roniciclib in activating apoptosis in MTC cells remain to be clarified.

Roniciclib arrested cells in the G2 phase in TT and DRO81-1 cells. Therefore, G2 phase arrest is likely one of the mechanisms of cytotoxicity in both MTC cell lines. The decreased levels of Aurora A may account for cell cycle arrest in G2 phase in MTC cells. Aurora A is a serine/threonine kinase that is required for G2 to M transition and mitotic progression [23, 24]. The effects of decreased levels of Aurora A may range from a failure in G2/M transition to mitotic arrest, depending on the magnitude of Aurora A affected. In this study, roniciclib induced cell accumulation in G2 phase in both cell lines. CDK1 activation is required for mitotic entry. Roniciclib has demonstrated to inhibit CDK1 activity [19]. Therefore, CDK1 inhibition is likely another mechanism accounting for G2 arrest in MTC cells. In addition, we found roniciclib decreased cell population in S phase that may lead to lower cell proliferation in TT and DRO81-1 cells.

Roniciclib treatment significantly repressed TT tumor growth. The anti-tumor effects are likely through both apoptosis induction and cell cycle inhibition because cleaved caspase-3 levels increased and Aurora A levels were decreased. Roniciclib treatment was able to decrease levels of proliferating cell nuclear antigen (PCNA) in TT tumors as compared with the untreated control tumor, indicating cell proliferation inhibition (Supplementary Figure 13).

Sorafenib therapy is suggested for patients with vandetanib- and cabozantinib-refractory MTC. However, some patients have experienced treatment failures with sorafenib therapy [26]. Novel strategies to improve outcomes of sorafenib treatment are needed. We found beneficial combination effects of roniciclib and sorafenib in the treatment of MTC tumors with promising safety profiles. This combination therapy regimen inhibited TT tumor growth significantly better than either drug treatment alone. The feasibility of combined roniciclib with vandetanib or roniciclib with cabozantinib in the treatment of MTC needs to be studied.

Our prior study revealed that roniciclib decreased survivin expression in anaplastic thyroid cancer tumors [28]. Survivin is a component of the chromosomal passenger complex that is involved in mitotic progression [29–31]. Depletion of survivin expression leads to inhibition of cell proliferation. We evaluated the effects of roniciclib on the expression of survivin in TT xenografts (Supplementary Figure 14). Survivin levels did not decrease after roniciclib treatment in TT tumors. The lower dose of roniciclib (1.0 mg/kg) treatment may not be sufficient to decrease survivin level in TT tumors, while higher doses of roniciclib therapy were able to decrease survivin levels in anaplastic thyroid cancer tumors (1.7 mg/kg). Another possibility for this discrepancy may be that survivin depletion by roniciclib treatment is cell-context dependent.

In conclusion, the CDK inhibitor, roniciclib, induces cytotoxicity in two MTC cell lines. *In vivo* study using TT xenograft tumors demonstrated the therapeutic efficacy and safety of roniciclib treatment. Combination therapy of roniciclib and sorafenib exhibited greater therapeutic efficacy than monotherapy in TT tumors. These data support clinical evaluation for the potential use of roniciclib in the treatment of patients with MTC.

## MATERIALS AND METHODS

### Cell lines

Two human MTC cell lines TT and DRO81-1 were evaluated [32–34]. TT was maintained in F12K. DRO81-1 was maintained in RPMI 1640 with sodium bicarbonate (2.0 g/L). Both media contained 10% fetal calf serum, 100,000 units/L penicillin and 100 mg/L streptomycin. All cells were maintained in a 5% CO_2_ humidified incubator at 37°C.

### Pharmacologic agents

Roniciclib and sorafenib were generous gifts from Bayer AG (Berlin, Germany). Roniciclib and sorafenib were dissolved in DMSO (Sigma) to a concentration of 10 mmol/L and stored at -30°C or -80°C until further use for *in vitro* experiments. For the *in vivo* studies, roniciclib was diluted in vehicle [40% poly(ethylene glycol) 300 (Sigma) and 60% water] to a concentration of 0.357 mg/mL before use. Sorafenib was dissolved in 50% Kolliphor EL (Sigma) and 50% ethanol (Sigma) to a concentration of 57.6 mg/mL and stored at -80°C. Sorafenib was further diluted with water to a final concentration of 14.4 mg/mL before *in vivo* use.

### Antibodies

Antibodies targeting caspase-3, cleaved caspase-3, Aurora A and PARP were purchased from Cell Signaling Technology. α-tubulin antibody was obtained from Sigma.

### Cytotoxicity assays

Cells were plated at 2 × 10^4^ cells per well in 24-well plates in 1 mL media. After overnight incubation, six serial two-fold dilutions of roniciclib, sorafenib or vehicle were added over a 4-day treatment course. Cytotoxicity was determined. Culture medium was removed and cells were washed with PBS and lysed with Triton X-100 (1.35%, Sigma) to release intracellular lactate dehydrogenase (LDH), which was quantified with a Cytotox 96 kit (Promega) at 490 nM by spectrophotometry (Infinite M200 PRO, Tecan). Each experiment was performed in triplicate, and the results are shown as the percentage of surviving cells determined by comparing the LDH of each sample relative to control samples, which were considered 100% viable. Dm on day 4 was calculated for each cell line using CompuSyn software [20, 21].

For combination therapy experiments, MTC cells were treated with roniciclib and sorafenib at a fixed dose ratio. Cells were incubated with vehicle, roniciclib, sorafenib, or combination therapy simultaneously for a 4-day course and cytotoxicity was measured. Six serial two-fold dilutions were examined at the following starting doses for TT and DRO81-1: roniciclib at 38.4 and 67.2 nmol/L, sorafenib at 7.2 and 19.2 mmol/L, respectively. The doses chosen were based on the Dm determined previously. Interactions between roniciclib and sorafenib were assessed by calculating the CI by Chou-Talalay equation.

### Apoptosis assessment

Caspase-3 activity was analyzed using a fluorometric assay kit (Abcam). Cells were plated at 1 × 10^6^ cells in 100-mm Petri dishes in 10 mL of media overnight. Roniciclib (100 nmol/L) or vehicle was added for 24 h. Adherent cells (5 × 10^5^) were collected, centrifuged, and lysed using 50 μL of lysis buffer on ice for 10 min, and incubated with DEVD-AFC substrate and reaction buffer at 37°C for 1.5 h. Caspase-3 activity was detected by spectrophotometry. Each condition was performed in duplicate.

The ability of roniciclib to induce sub-G1 apoptotic cells was studied using flow cytometry. Cells were plated at 4 × 10^5^ cells per well in 6-well plates in 2 mL media overnight. Roniciclib (100 nmol/L) or vehicle was added and incubated for 24 h and 48 h. Floating cells and trypsinized adherent cells were collected, washed with PBS, fixed with cold 70% ethanol and incubated with RNase A (100 μg/mL; Sigma) and propidium iodide (5 μg/mL; Sigma) at 37°C for 15 min. Apoptotic sub-G1 cells were assessed by DNA content detected by flow cytometry (BD FACScalibur Flow Cytometer, BD Biosciences). Each condition was performed in triplicate.

Early apoptosis was measured by Annexin V-Alexa Fluor 488 and PI staining kit (Invitrogen). Cells were plated at 4 × 10^5^ cells per well in 6-well plates in 2 mL of media overnight and treated with roniciclib (100 nmol/L) or placebo for 24 h. Adherent cells were collected, washed with PBS and incubated with Annexin V-Alexa Fluor 488 and PI at room temperature in the dark for 15 min according to the manufacturer's protocol. Early apoptotic cells (Annexin V-positive, PI-negative) were detected by flow cytometry (BD FACScalibur Flow Cytometer, BD Biosciences). Each condition was performed in triplicate.

### Cell cycle assessment

The effects of roniciclib on cell cycle progression were evaluated. Cells were plated at 4 × 10^5^ cells per well in 6-well plates in 2 mL of media overnight. Roniciclib (100 nmol/L) or vehicle was added and incubated for 24 h, after which adherent cells were collected, and samples were prepared as described above for sub-G1 apoptosis. Cell cycle distribution was assessed by DNA content detected by flow cytometry (BD FACScalibur Flow Cytometer, BD Biosciences). Each condition was performed in triplicate.

### Immunofluorescence microscopy

The effect of roniciclib on mitotic progression was evaluated using confocal microscopy. Thyroid medullary cancer cells were plated at 1 × 10^5^ cells in 4-well culture slides in 1 mL of media overnight. Cells were treated with roniciclib (100 nmol/L) or placebo for indicated periods, washed with PBS, fixed in 4% paraformaldehyde (Sigma) for 15 min at room temperature, washed with PBS, permeabilized with 0.1% Triton X-100 (10 min, room temperature), washed with PBS. Cells were then incubated with primary mouse α-tubulin antibody (1:1000) at 4°C overnight, washed with PBS and incubated with secondary Alexa Fluor 488-conjugated goat anti-mouse antibody (1:1000; Life Technologies) for 25 min at 37°C, washed with PBS, incubated with 4′,6-diamidino-2-phenylindole (DAPI; 0.2 μg/mL, Invitrogen) for 10 min at room temperature, washed with PBS, and covered with Vectashield mounting medium (Vector Laboratories). Images were captured with Leica TCS SP8 X confocal microscopy (Leica Microsystems). Chromosomes were examined to identify mitotic cells.

The expression of cleaved caspase-3 and Aurora A was evaluated using immunofluorescence microscopy. Roniciclib (100 nmol/L) or placebo treated MTC cell samples were prepared as described above. Cells were then incubated with primary rabbit cleaved caspase-3 antibody (1:400), rabbit Aurora A antibody (1:200), and mouse α-tubulin antibody (1:1000) at 4°C overnight, washed with PBS and incubated with secondary Alexa Fluor 633-conjugated goat anti-rabbit antibody (1:1000; Invitrogen) and Alexa Fluor 488-conjugated goat anti-mouse antibody (1:1000; Life Technologies) for 25 min at 37°C, washed with PBS, counterstained with DAPI, washed with PBS and covered with mounting medium. Images were acquired using Leica TCS SP8 X confocal microscopy.

### Flank xenograft tumor therapy

Female 7-10 weeks old athymic nude mice from the National Laboratory Animal Center, Taiwan, were anesthetized with an intraperitoneal injection of 2% 2,2,2-tribromoethanol (200 μL/mouse; Sigma) before implantation of thyroid cancer cells. TT flank tumors were established by injecting 1 × 10^6^ cells in 100 μL of ECM gel (Sigma) into the subcutaneous flanks of nude mice.

For monotherapy with roniciclib, mice bearing TT xenograft tumors received oral administration of vehicle or roniciclib (1.0 mg/kg) twice a day for three cycles of 3-day on and 3-day off therapy. For roniciclib and sorafenib combination therapy, mice bearing TT tumors received placebo, roniciclib (1.4 mg/kg), sorafenib (25 mg/kg), or simultaneous combination treatment once a day for three cycles of 4-day on and 3-day off treatment. Tumor dimensions were serially measured with electronic calipers, and the volumes were calculated by the following formula: a × b^2^ × 0.4, where a represents the largest diameter and b is the perpendicular diameter. The body weight of each animal was followed as a marker of toxicity.

This study was performed in accordance with the recommendations in the Guide for the Care and Use of Laboratory Animals of the Chang Gung Memorial Hospital, and the protocol was approved by the Committee of Laboratory Animal Center at Chang Gung Memorial Hospital, Linkou (permission No: 2013121401). Animals were given *ad libitum* access to food and water.

### Western blot analysis

TT and DRO81-1 cells were plated at 1 × 10^6^ cells in 100-mm Petri dishes in 10 mL of media overnight and treated with roniciclib at 100 nmol/L for the indicated periods. Cell pellets were dissolved in radio-immunoprecipitation assay buffer and protease inhibitor cocktail, vortexed and clarified by centrifugation. Total protein was separated by electrophoresis on 12% Tris-HCl gels, transferred to polyvinylidene difluoride membranes, blocked and exposed to primary antibodies followed by a secondary antibody conjugated to horseradish peroxidase. Signals were developed using an enhanced chemiluminescence kit (PerkinElmer).

Tumor levels of cleaved caspase-3, cleaved PARP and Aurora A were evaluated in mice treated with oral administration of roniciclib by western blot analysis. At indicated periods, animals were euthanized with carbon dioxide, and the tumors were harvested, mixed with protein extraction buffer (GE Healthcare), homogenized, and sonicated on ice. After centrifugation, clarified supernatants were aliquoted and stored at -80 °C for western blotting as described above. The ratios of cleaved caspase-3, cleaved PARP and Aurora A to α-tubulin at each time point were calculated. Relative expression was analyzed using the control value as reference.

### Statistical analysis

Comparisons were performed when appropriate, using two-sided Student's *t* tests (Excel, Microsoft). P < 0.05 was considered statistically significant. Results are expressed as mean ± SE.

## SUPPLEMENTARY MATERIALS FIGURES


