# Supplementary material for: Activity of roniciclib in medullary thyroid cancer
Source: Oncotarget. 2018 Jun 15;9(46):28030–41. doi: 10.18632/oncotarget.25555 (PMC6021325; doi:10.18632/oncotarget.25555)
Supplement: Supplementary file 1 [file oncotarget-09-28030-s001.pdf]

Activity of roniciclib in medullary thyroid cancer

SUPPLEMENTARY MATERIALS

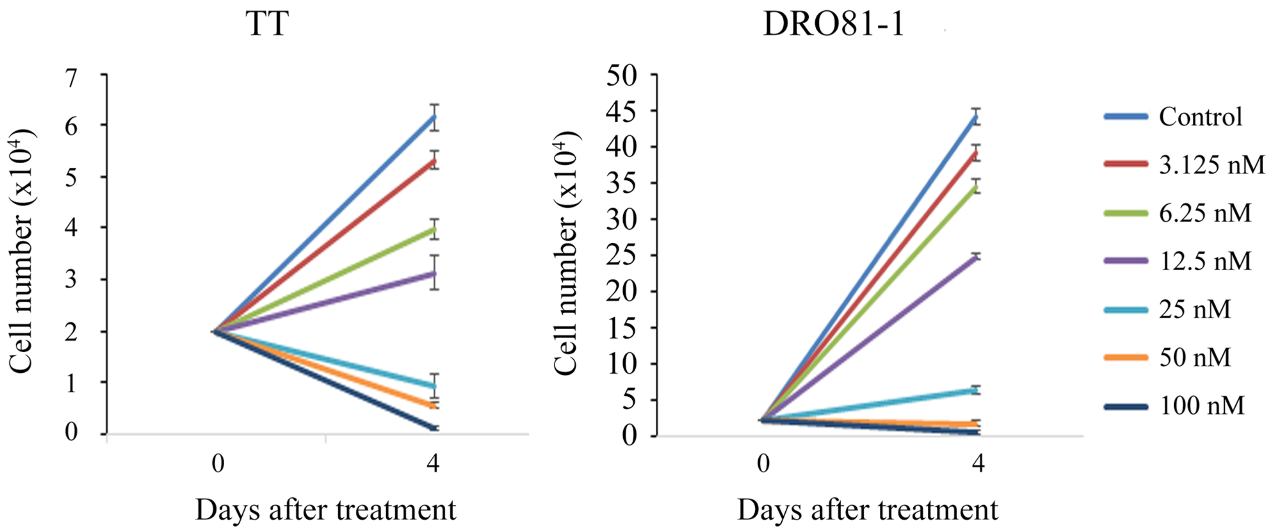

Supplementary Figure 1: Roniciclib inhibits cell proliferation in MTC cell lines. TT and DRO81-1 cells were plated at 2×10^4 cells per well in 24-well plates in 1 mL media. After an overnight incubation, six serial two-fold dilutions of vehicle or roniciclib were added at the starting dose of 100 nmol/L. Viable cells were counted using the microscope after a 4-day treatment.

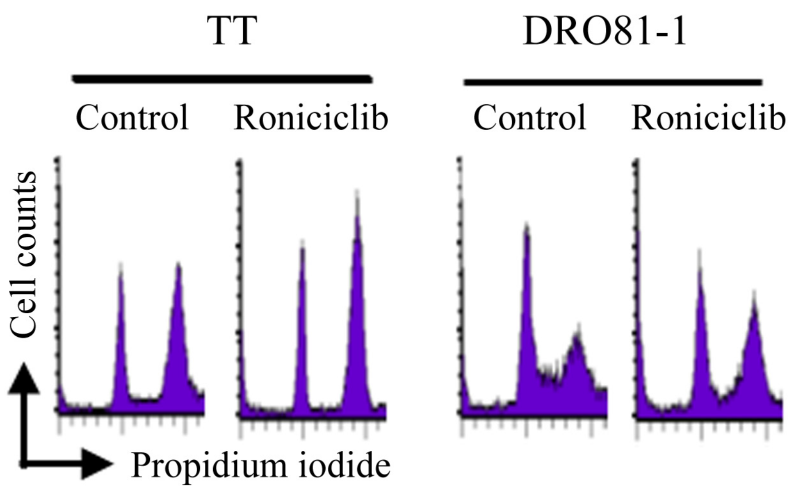

Supplementary Figure 2: The effects of roniciclib on sub-G1 apoptosis in MTC cells. DNA content analysis using flow cytometry was performed to identify sub-G1 apoptotic cells in TT and DRO81-1 cells treated with placebo or roniciclib (100 nmol/L) for 24 h.

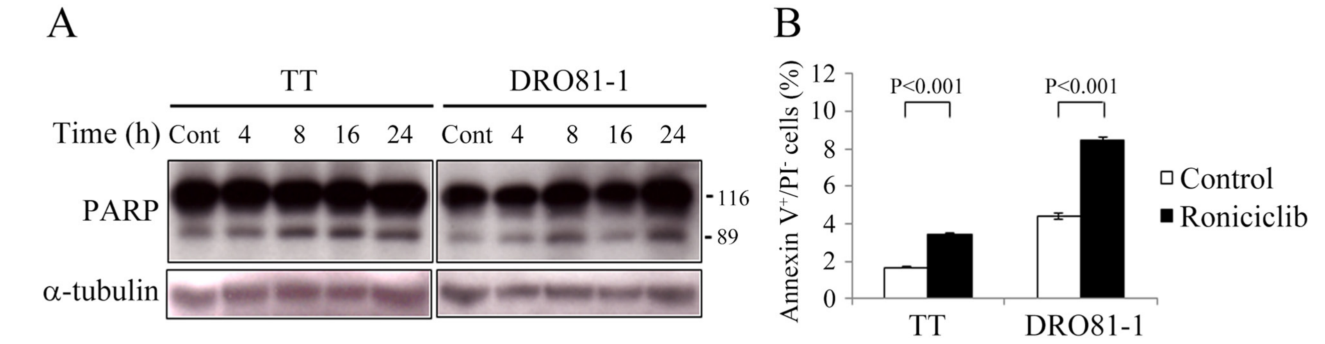

Supplementary Figure 3: Roniciclib induces early apoptosis in MTC cells. (A) TT and DRO81-1 cells were treated with roniciclib (100 nmol/L) for indicated period and the level of cleaved PARP was analyzed using western blot. Roniciclib increased the expression of cleaved PARP by 8 h in two MTC cell lines. **(B)** The percentages of cells in early apoptosis (Annexin V positive/PI negative cells) were assessed using flow cytometry after treatment with placebo or roniciclib (100 nmol/L) for 24 h in TT and DRO81-1 cell lines. Roniciclib significantly increased the proportion of early apoptotic cells in these MTC cell lines.

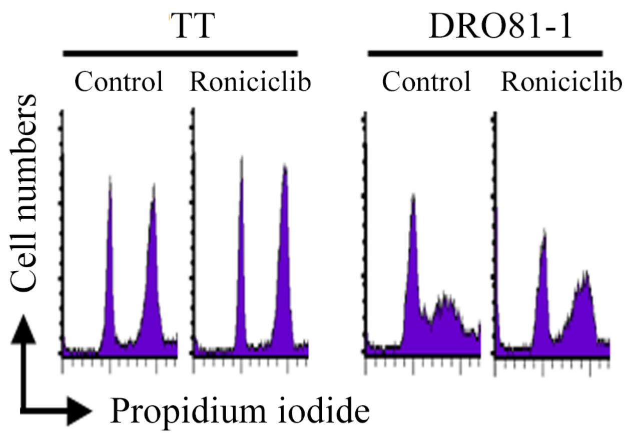

Supplementary Figure 4: The effects of roniciclib on cell cycle distribution in MTC cells. Cell cycle analysis using flow cytometry was performed to evaluate DNA content in TT and DRO81-1 cells treated with placebo or roniciclib (100 nmol/L) for 24 h.

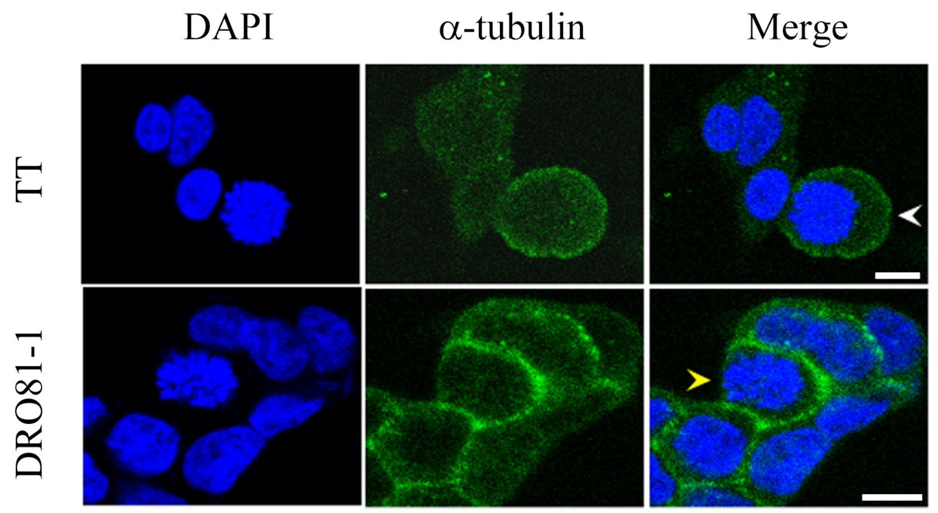

Supplementary Figure 5: Chromosomal appearance of TT and DRO81-1 cells in mitosis. Cells were plated overnight and treated with placebo for 24 h and stained with fluorescent antibodies against DAPI (blue) and α-tubulin (green). A TT cell in prophase (white arrowhead) and a DRO81-1 cell in prometaphase (yellow arrowhead) were identified.

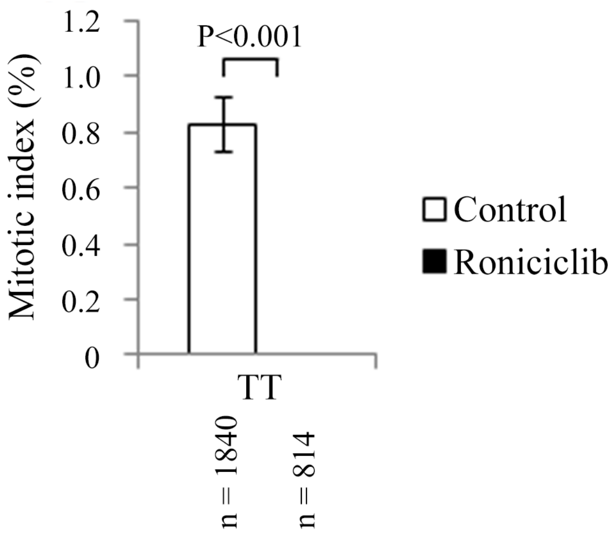

Supplementary Figure 6: Roniciclib inhibits mitotic entry in TT cells. The proportion of TT cells in mitosis was assessed after treatment with roniciclib (100 nmol/L) or placebo for 48 h. Cells were stained with DAPI and chromosome characteristics were evaluated using immunofluorescence confocal microscopy. Mitotic index was assessed with a minimum of 814 cells and counted from at least ten different fields for each condition. Roniciclib significantly decreased the proportion of cells in mitosis in TT cell lines.

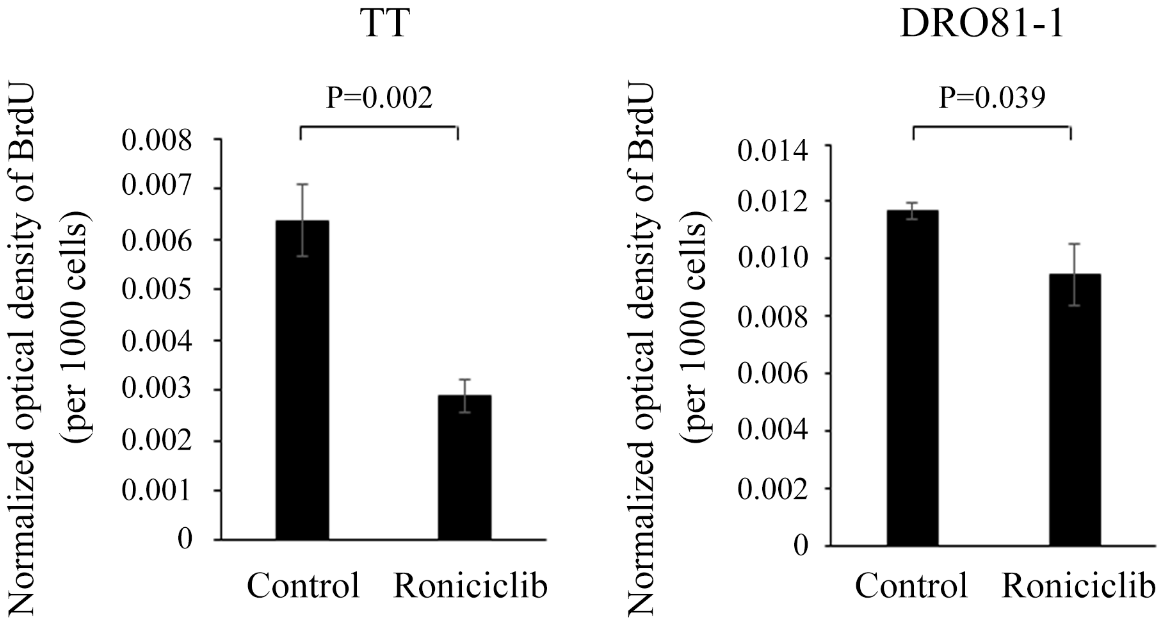

Supplementary Figure 7: The effects of roniciclib on BrdU uptake in MTC cells. Cells were plated at 4×10^3 (TT) and 2×10^3 (DRO81-1) in 96-well plates in 100 µL media. After overnight incubation, roniciclib (100 nmol/L) and placebo were added. Then, BrdU was added and cells were incubated for 24 h (TT) and 16 h (DRO81-1). BrdU uptake was assessed using a colorimetric assay kit (Cell Signaling Technology). Roniciclib significantly decreased BrdU uptake in TT and DRO81-1 cells when compared with control treatment.

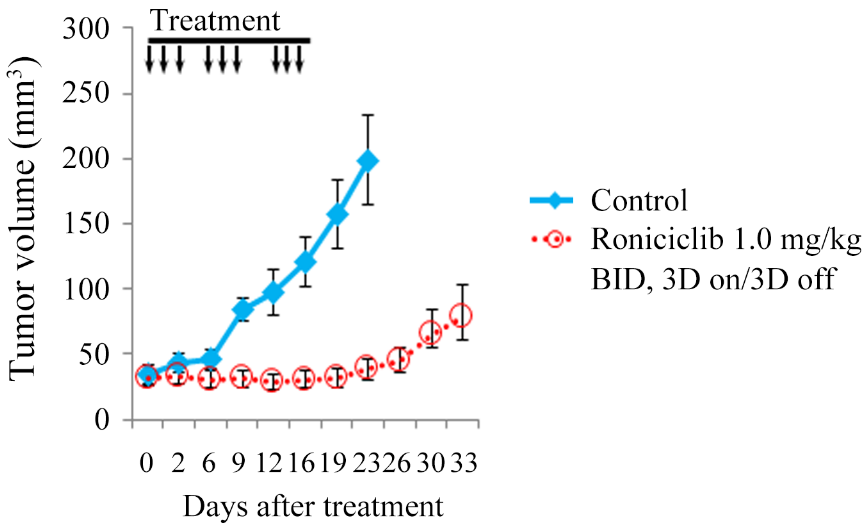

Supplementary Figure 8: Roniciclib inhibits subcutaneous xenograft growth of TT tumor. The therapeutic efficacy of roniciclib was evaluated in mice bearing TT flank tumors. Serial oral gavage of roniciclib (1.0 mg/kg) retarded TT tumor growth compared with the control group. Arrow, roniciclib or placebo treatment.

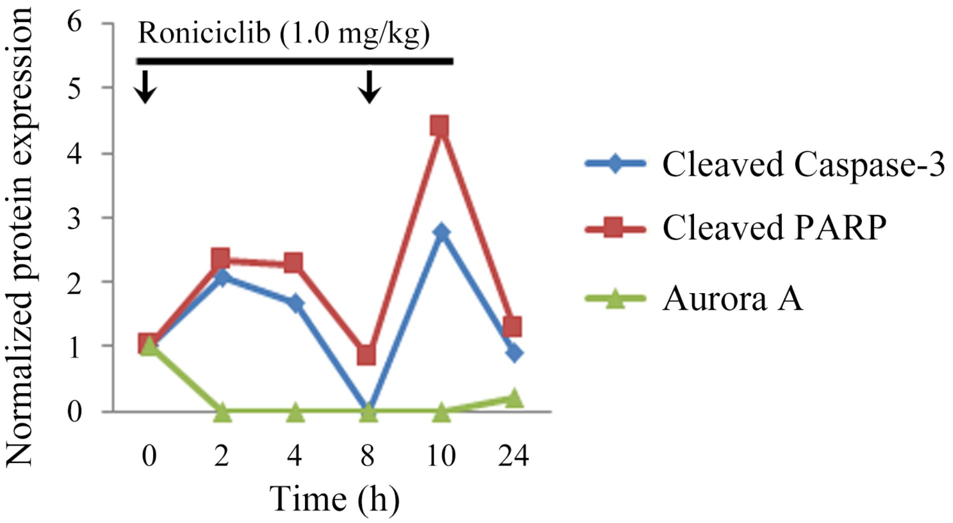

Supplementary Figure 9: Effects of roniciclib on the expression of cleaved caspase-3, cleaved PARP and Aurora A in murine MTC xenograft tumor. Oral administration of roniciclib (1.0 mg/kg) twice a day decreased Aurora A level by 2 h, and the effect persisted till 24 h in TT tumors. Cleaved caspase-3 and cleaved PARP were increased by 2 h, and the stimulatory effects were diminished by 8 h.

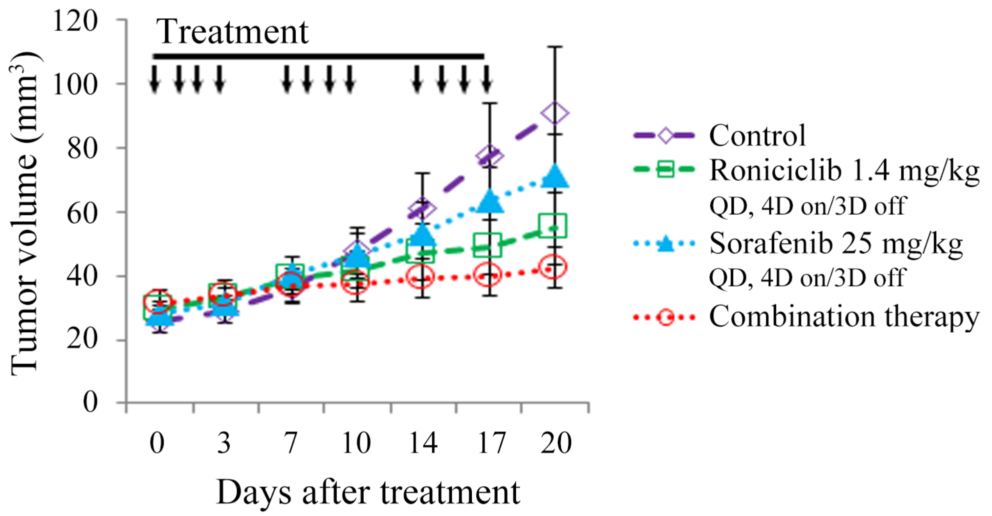

Supplementary Figure 10: Combination of roniciclib and sorafenib therapy in TT xenograft tumor model. TT flank tumors were established in nude mice and treated with oral gavage of placebo, roniciclib (1.4 mg/kg), sorafenib (25 mg/kg) and combination therapy once a day for three cycles of 4-day on and 3-day off therapy. Tumor growth was lower for mice treated with combination therapy compared with single modality treatment or control. Arrow, placebo, roniciclib, sorafenib and combination treatment.

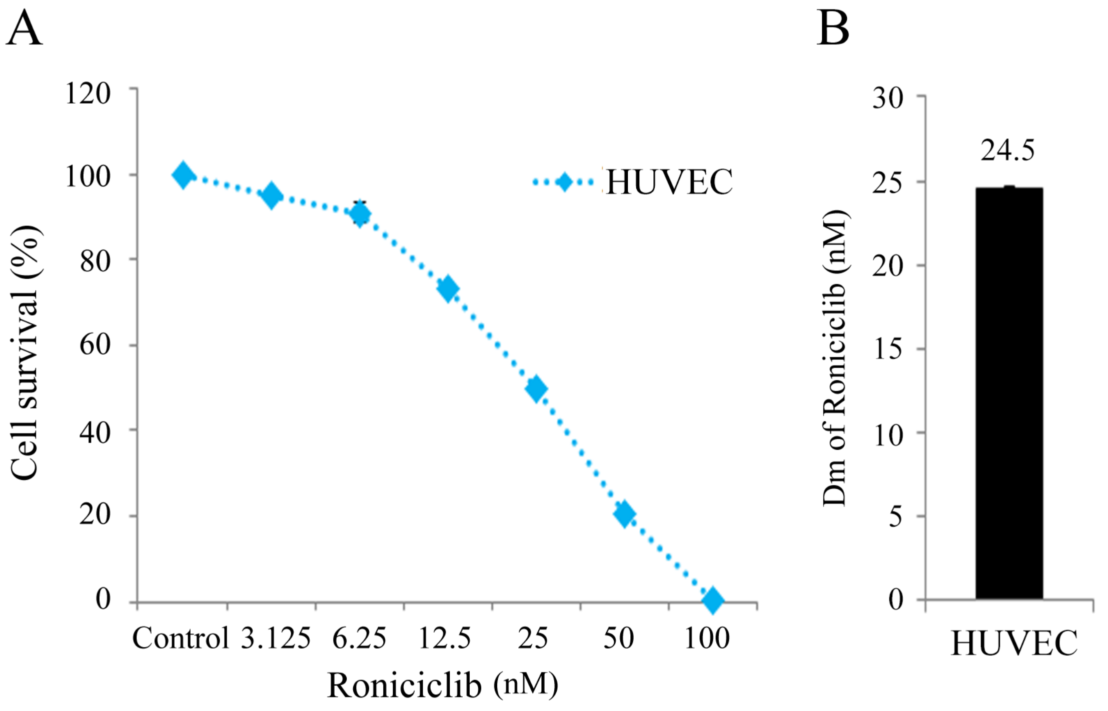

Supplementary Figure 11: Cytotoxicity of roniciclib in non-cancerous human umbilical vein endothelial cells (HUVEC). **(A)** Cytotoxicity was evaluated in HUVEC (Thermo Fisher Scientific Inc.) treated with a series of six two-fold dilutions of roniciclib starting from 100 nmol/L. Dose-response curve was obtained on day 4 using LDH assays. **(B)** Median-effect dose (Dm) of roniciclib on day 4 was calculated for HUVEC using CompuSyn software.

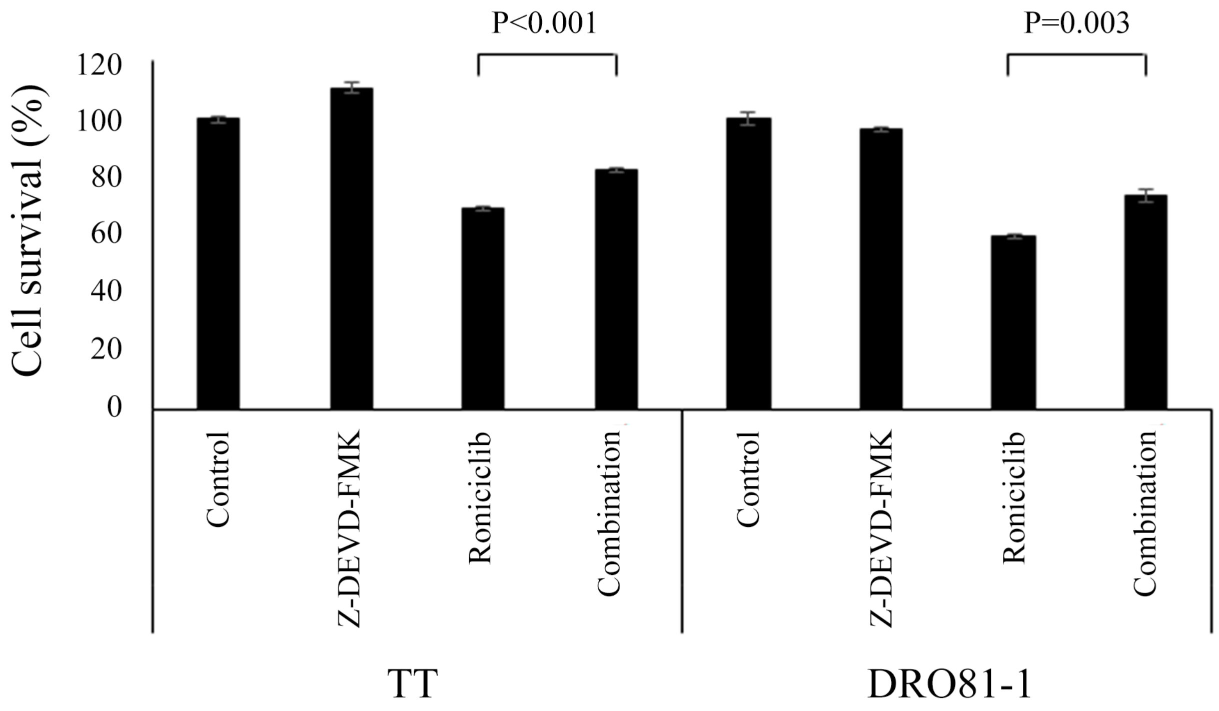

Supplementary Figure 12: Inhibition of caspase-3 activity decreases the cytotoxicity of roniciclib in MTC cells. TT and DRO81-1 cells were pretreated with a caspase-3 inhibitor, Z-DEVD-FMK (Biovision, 1:1000) or placebo for 1 h, followed by incubation with roniciclib (100 nmol/L) for 24 h. With the presence of Z-DEVD-FMK, more cells were viable in TT (from 69.4 ± 0.8% to 82.4 ± 0.8%, P < 0.001) and DRO81-1 (from 59.9 ± 0.8% to 73.8 ± 2.1%, P = 0.003).

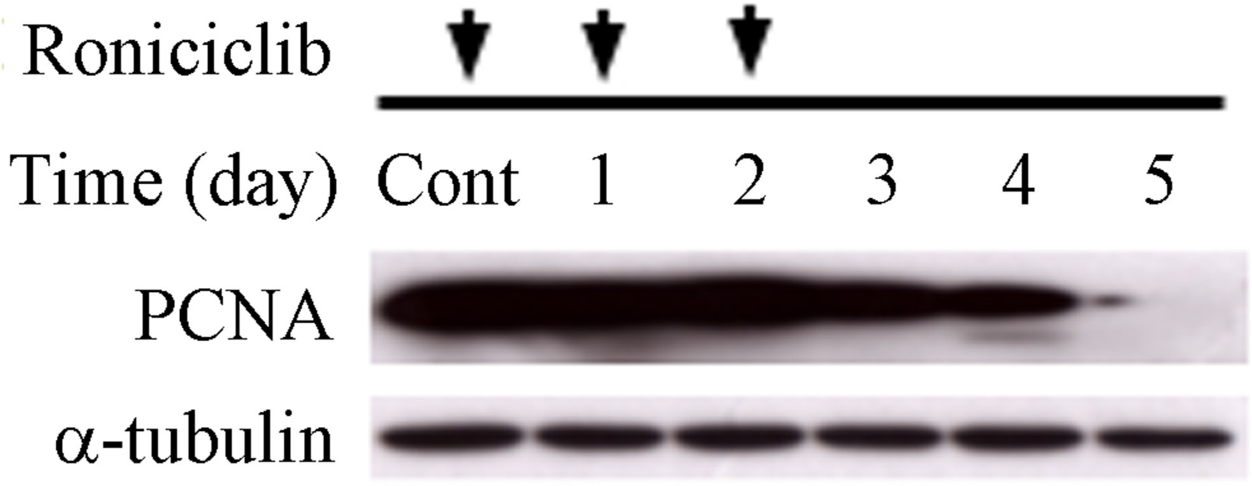

Supplementary Figure 13: Effects of roniciclib on the expression of PCNA in murine MTC xenograft tumors. Oral administration of roniciclib (1.0 mg/kg) twice a day for 3 days decreased PCNA expression by 4 days in TT tumors as compared with control untreated tumor. Arrow, roniciclib treatment.

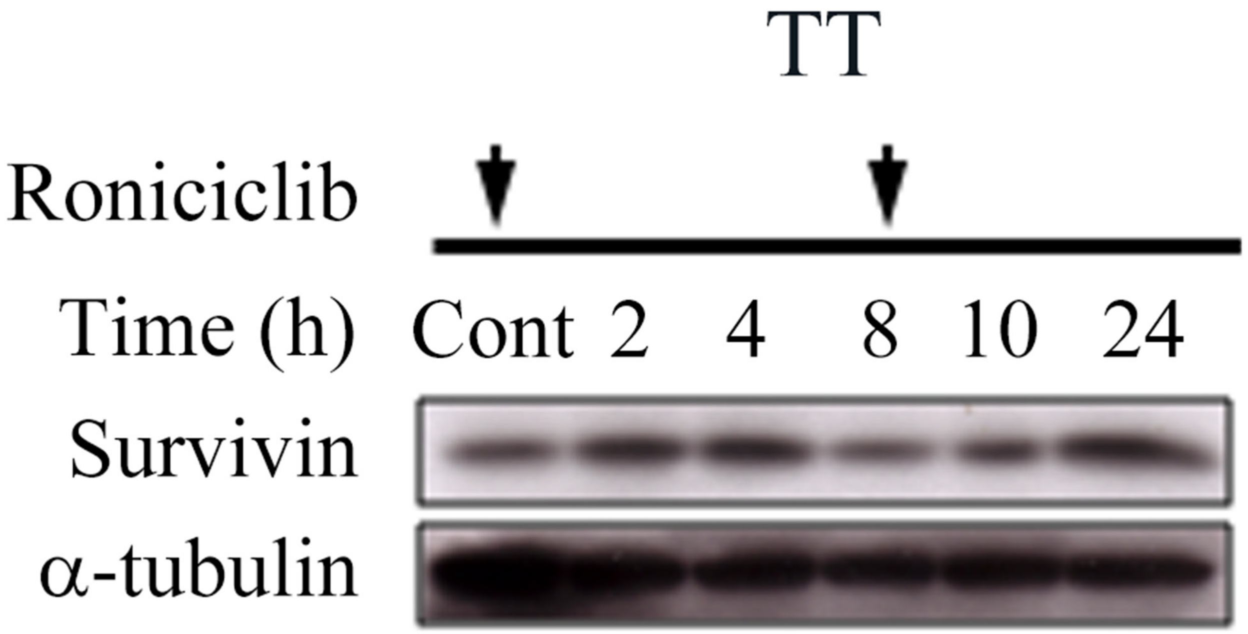

Supplementary Figure 14: Effects of roniciclib on the expression of survivin in murine MTC xenograft tumor. Oral administration of roniciclib (1.0 mg/kg) did not decrease survivin expression in TT tumors as compared with control untreated tumor. Arrow, roniciclib treatment.
